# Complete Breast Cancer Detection and Monitoring System by Using Microwave Textile Based Antenna Sensors

**DOI:** 10.3390/bios13010087

**Published:** 2023-01-04

**Authors:** Dalia N. Elsheakh, Rawda A. Mohamed, Omar M. Fahmy, Khaled Ezzat, Angie R. Eldamak

**Affiliations:** 1 Department of Electrical Engineering, Faculty of Engineering and Technology, Badr University in Cairo, Badr City 11829, Egypt; 2 Microstrip Department, Electronics Research Institute, Nozha, Cairo 11843, Egypt; 3 Electronics and Communications Engineering Department, Faculty of Engineering, Ain Shams University, Cairo 11517, Egypt

**Keywords:** wearable, breast cancer, textile antenna sensor, smart bra, coplanar waveguide monopole antenna specific absorption rate (SAR), machine-learning algorithms (MLA), ultra-wide bandwidth (UWB)

## Abstract

This paper presents the development of a new complete wearable system for detecting breast tumors based on fully textile antenna-based sensors. The proposed sensor is compact and fully made of textiles so that it fits conformably and comfortably on the breasts with dimensions of 24 × 45 × 0.17 mm^3^ on a cotton substrate. The proposed antenna sensor is fed with a coplanar waveguide feed for easy integration with other systems. It realizes impedance bandwidth from 1.6 GHz up to 10 GHz at |S_11_| ≤ −6 dB (VSWR ≤ 3) and from 1.8 to 2.4 GHz and from 4 up to 10 GHz at |S_11_| ≤ −10 dB (VSWR ≤ 2). The proposed sensor acquires a low specific absorption rate (SAR) of 0.55 W/kg and 0.25 W/kg at 1g and 10 g, respectively, at 25 dBm power level over the operating band. Furthermore, the proposed system utilizes machine-learning algorithms (MLA) to differentiate between malignant tumor and benign breast tissues. Simulation examples have been recorded to verify and validate machine-learning algorithms in detecting tumors at different sizes of 10 mm and 20 mm, respectively. The classification accuracy reached 100% on the tested dataset when considering $\left|S_{21}\right|$ parameter features. The proposed system is vision as a “Smart Bra” that is capable of providing an easy interface for women who require continuous breast monitoring in the comfort of their homes.

## 1. Introduction

According to the World Health Organization (WHO), the most common cancer detected among women is breast cancer. The incidence rate of women in the world reached 25.2%, and the number of cases diagnosed with breast cancer worldwide reached about 1.7 million [1,2]. According to the American Cancer Society in 2019, more than 40,000 women died of breast cancer, and more than 260,000 new cases of breast cancer would be diagnosed in the United States in 2020 [1,2]. More than 22,000 new cases of breast cancer are diagnosed annually in Egypt, where it accounts for 33% of all female cancer cases [3]. In 2018, there were about 134,632 new cancer cases and 89,042 cancer-related deaths in Egypt only. Detecting breast cancer early enough is key to successful treatment, with a five-year survival rate of over 90% [4,5]. Therefore, regular check-ups and early detection of breast cancer is very important.

The current techniques for breast imaging are ultrasound, magnetic resonance imaging, and the most famous technique, X-ray mammograms [6,7]. Research has shown that more women in their forties suffer harm from starting regular mammograms than older women. Mammographic findings revealed that 1212 of the 10,000 women examined turned out to be a false positive in their forties [6,7]. Adverse effects of mammography include excessive treatment and unnecessary follow-up tests, other than the psychological harm caused by false positive test results. Ultrasonic pulses are created by placing a transducer on the skin, which transmits ultrasound pulses to the breasts and listens for their echoes inside the breasts. Although this procedure is painless and radiation-free, ultrasonography has a low spatial resolution and cannot differentiate between dangerous and benign tumors. On the other hand, an MRI creates images of the inside of the body using radio waves and powerful magnetic fields [8,9]. Additionally, a contrast fluid is injected when it is used on the breasts (soft tissue) to obtain a crisper image. Prior to surgery, the examination is evaluated using magnetic resonance imaging. The costs associated with MRI exams are high, and incorrect placement of the breasts might result in a missed diagnosis [9,10].

**Figure 1:**
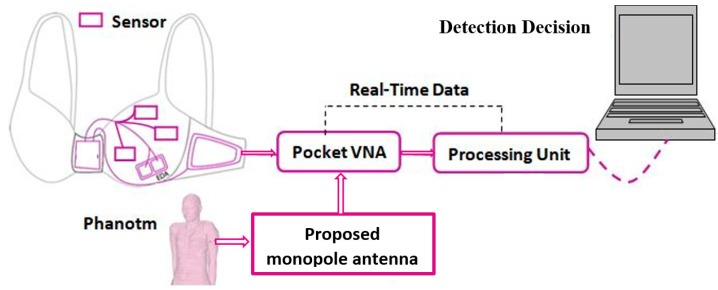
The proposed breast cancer detection system as a “Smart Bra”.

Recently, microwave imaging and detection systems offered a great alternative in terms of resolution, safety, pain-free, as well as low cost and less scanning time. Sensing using microwaves mainly relies on detecting differences in electrical properties between normal and cancerous tissues in the breasts [11,12,13,14,15,16,17,18,19,20,21,22,23,24,25,26,27,28,29,30,31]. Several studies reported a significant contrast in the range of 1:2.3 to 1:10 between dielectric properties of healthy and cancerous breast tissues [21,24,25,26,31]. Thus, the idea of developing wearable detection systems using microwaves is more eminent and will be capable of detecting tissues with breast cancer [21,24,25,26,31]. One of the most important elements in the microwave detection system is the antenna used to transmit/or receive electromagnetic energy. Many antennas have been designed for breast cancer detection, such as horn antenna [12], CPW antenna [13], 3D antenna [14], microstrip antenna [15], dielectric resonator antenna (DRA) [16], and monopole antenna. Thus, developing textile-based antennas as wearable antenna sensors is emerging in the literature [17,18,19,20,21,22,23]. Many textile-based antennas are reported in the literature for various applications and specifically developed by our group in [24,25]. This study was conducted towards developing wearable microwave imaging systems for breast cancer detection and monitoring as shown in Figure 1.

For data classification and interpretation, researchers used machine-learning techniques to improve the performance of microwave imaging systems [26,27,28,29,30,31]. In [32], the detection of breast lesions based on radar techniques have utilized machine-learning techniques for detecting lesions. Microwave signals of a generated 3D tumor model created a dataset of features to train a support vector machine (SVM) as well as logistic regression classifiers in order to detect malignant tumors. In [33], clinical data of a microwave mammogram were collected to train a classifier that detects breast lesions. The authors achieved a classification accuracy of 85%; however, they detected the breast tumor with $S_{11}$ parameters only, which limits the classification accuracy. Different machine-learning algorithms have been utilized for classification task on S-parameters of the proposed antenna sensors. These classification algorithms are applied on both $S_{11}{\;\mathrm{and}\;S}_{21}$ of microwave antenna sensors. The classification algorithms are trained and tested to detect tumors of sizes 10 mm and 20 mm, respectively. These examples show the effect of $S_{21}$ as well as the tumor size on the classification accuracy.

This paper proposes a wearable flexible system for breast monitoring and screening. This is realized by developing antenna-based sensors using only textiles. The proposed sensors use microwave signal for breast screening. The given system provides a broadband performance in the ISM band from 1.8 GHz to 10 GHz. The antenna-based sensor is composed of a monopole antenna with an overall compact size of 24 × 45 mm^2^. Performance of the monopole antenna allows integration into wearables unlike similar work in the literature using end-fire Vivaldi antennas. Most systems relying on Vivaldi antennas will require patients to position themselves into the scanning system. Moreover, specific absorption rate (SAR) is measured and recorded for the proposed antenna-based sensors. The proposed antenna-based sensor realized an improved low SAR compared to the standard rated value, which validates its safety as a single element or array in the proposed wearable system. Moreover, phantoms for the breast and tumor have been fabricated as well in this paper to validate the operation of the proposed antenna-based textile sensors. To complement the proposed system, detection algorithms based on machine learning have been developed and tested as well using S-parameters of the proposed antenna-based sensors. The detection results validate the potential of the proposed system for breast cancer detection, monitoring, as well as imaging.

## 2. Materials and Methods

### 2.1. Wearable Breast Cancer Monitoring System

Electronic devices or smart sensors (antennas) can now be worn directly on the body by using wearable technologies. It has a lot of benefits, including constant health monitoring with minimal energy use. Due to its low-cost, low-profile, non-ionizing, and non-intrusive characteristics, wearable microwave detection and imaging systems have gained popularity as a tool for long-term disease assessment or routine monitoring. It is crucial to employ soft, pleasant materials while creating wearable technology. The proposed detection and monitoring system for the breasts is visioned in Figure 1 as a “Smart Bra”.

It proposes a new comfortable wearable system that will help women to receive regular breast cancer screening safely, specifically young women with dense breasts, using microwave imaging. The prospective system is planned to use textiles for fabricating antenna-based sensors. Microwave breast imaging has the potential to replace or act as an additional tool to the standard X-ray mammography for detecting breast cancer. The system will provide a graphical user interface connected to the system through a control unit as shown in Figure 1. The results from microwave breast monitoring systems are very promising so far due to the difference in dielectric properties between malignant tissue and normal breast tissue. Thus, electromagnetic sensors’ optimization is critical to design new antenna-based sensors to enhance detection results focusing on higher gain and broader bands while keeping the antenna size. Optimizing the size of the single element antenna will allow building larger arrays with more collected information of the scattered signal for a successful detection. Realizing a wide bandwidth will help in constructing a high-resolution image and reducing the distortion in transmission of short-duration pulses. Many efforts are conducted to investigate new sensors with characteristics suitable to satisfy the competing requirements of the microwave breast imaging and detection systems.

### 2.2. Antenna Sensor Fabrication Technologies and Materials

Multiple ways exist to manufacture textile-based antennas acting as antenna sensors, where performance depends on the material used, fabrication technique, and the substrate properties [29,34,35,36,37]. The antenna sensor with a thin layer of uniform metallization is deployed on substrates. To encounter for the given application, cotton substrates are chosen based on the study conducted previously in [24,25]. Cotton is used as substrate material in the designed proposed antennas with dielectric constant ε = 1.9, loss tangent = 0.04, and thickness = 2 mm [24,25]. Cotton as a substrate is a good alternative for the fabrication of the wearable antenna sensors as it provides comfort, absorption of human sweat, ease of fabrication, and cost efficiency. Moreover, cotton substrates are biodegradable and environmentally friendly [34].

A thin layer of uniform metallization layer can form the structure of the given antenna sensor by using either copper tape, embroidered conductive threads, conductive textile, or using inkjet screen printing on a non-conductive textile acting as the substrate [35]. Copper tape was not chosen as it is not ideal for long-term usage as bending and humidity may cause it to detach from the substrate. Also, the conductive threads that are embroidered to the textile non-conductive substrate to form the antenna with low radiation efficiency and it is affected by the washing process. Computer-aided embroidery machines help in constructing complex antenna structures with neither huge time consumption nor inaccuracy [36]. However, multiple parameters control the efficiency and the performance of the embroidered antenna. Firstly, the DC resistance of the conductive threads can affect the radiation efficiency along with the losses inside the substrate [37]. The DC resistance of the conductive threads are depend on both a function of the material type and the stitch type, as well as the tension or the stretching force.

**Figure 2:**
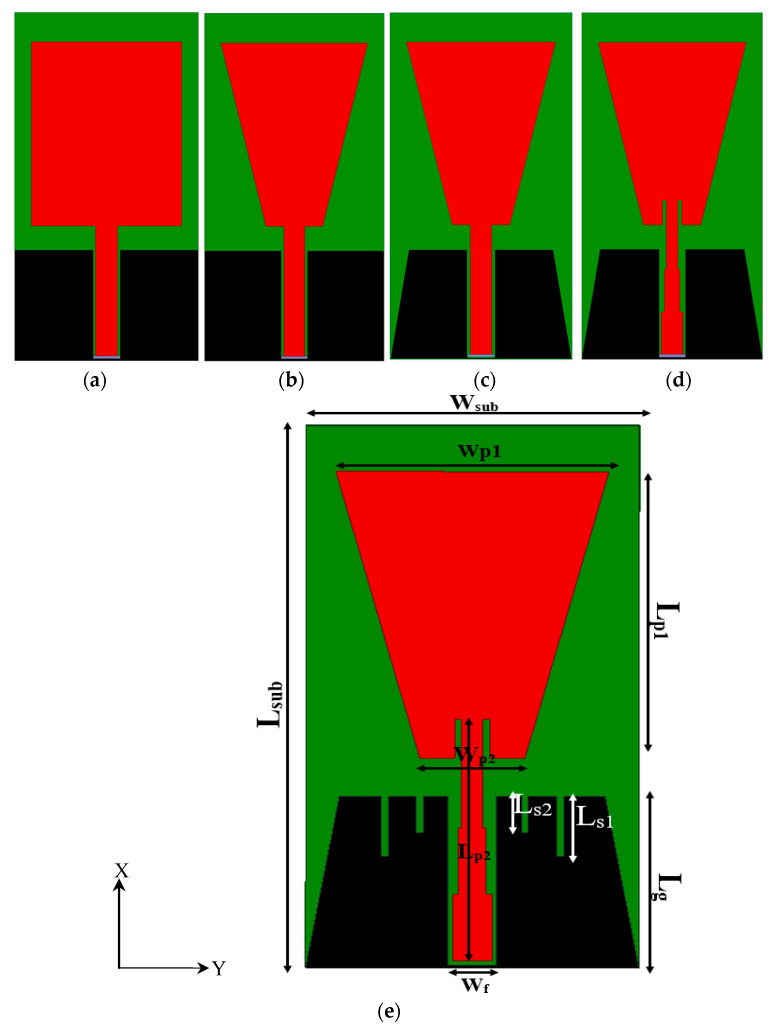
(**a**–**d**) The design steps of CPW-based monopole antenna and (**e**) final design of CPW-based monopole antenna.

In this paper, the proposed antenna sensors will be fabricated using two technologies for metallization. The first technology uses a flexible Roger substrate fabricated by using photographic technique, while the second uses conductive fabric attached on the cotton substrate using glue [35,36,37]. The second technology uses conductive fabric. In [38], four different methods were used for attaching the conductive fabric. The sewing method is very slow and requires accuracy to not cause any shorting during the sewing procedure. This technique of manufacturing flexible antenna is more tolerant and practical compared to using copper tape [38]. On the other hand, conductor fabrics such as nylon (Nora-Dell-CR Fabric), conductive polymers (PANI/CCo), ShieldIt, and Coatex have constant sheet resistance per square and are shaped manually using regular cutting tools. The conductive fabric with adhesive layer is then integrated into the dielectric fabric by ironing or by sewing directly onto the dielectric fabric. Moreover, heat is totally avoided in connecting the SMA connector to the textile-based wearable antenna. Instead, conductive glue, known as an epoxy adhesive, is used to attach connectors to all proposed antenna sensors for characterization and measurements. The proposed antenna is symmetric with respect to its axis as shown in Figure 2. The proposed design starts as a simple rectangular patch monopole antenna with coplanar waveguide fed as shown in Figure 2a, which is developed to the monopole antenna trapezoidal shape as shown in Figure 2b. A modified ground plane is used as shown in Figure 2c. Two inset stubs with different impedance transmission line values are added as shown in Figure 2d. Finally, different stub lengths are added to the feed line, and the dimensions of the proposed monopole are optimized as shown in Table 1. The final design of the proposed antenna sensor with all design parameters labelled is shown in Figure 2e.

### 2.3. Phantom Fabrication Materials

**Figure 3:**
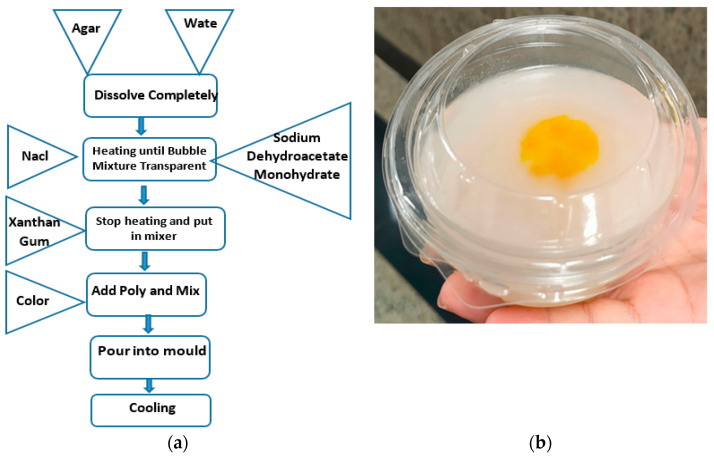
Breast and tumor models fabrication: (**a**) fabrication flow chart and (**b**) breast model with tumor cells in the middle.

Many types of mixtures are reported in the literature to replicate breast and tumor phantoms [39,40,41,42]. In the proposed work, as shown in Figure 3a, the recipe reported in [43] was adopted in the fabrication of both the breast and tumor models. For the breast models, 4.5 g of agar is dissolved in 50 mL of distilled water, 150 mL corn oil, and 30 mL neutral detergent. Next, sodium chloride and sodium ethate monohydrate are added to the mixture. After the solution is dissolved, it is heated up to 80 °C until bubbles begin to form, and the mixture becomes transparent. At that stage, the heat is turned off and xanthan gum is added to the solution and mixed. Finally, polyethylene powder is added to obtain a homogeneous mixture. The mixture is poured into a mold suitable for the anatomical structure. On the other hand, the tumor model is prepared in the same way but with different proportions of 100 mL deionized tri-distilled water, 60 mL ethanol, 1 g NaCl, and 1.5 g agarose [43]. For all materials used, a little yellow color is used to distinguish the tumor model as shown in Figure 3b. All models were kept in the refrigerator to solidify. Figure 3a presents the flow chart of the fabrication of the breast and tumor models as well as the fabricated breast model with tumor inserted in the middle.

**Figure 4:**
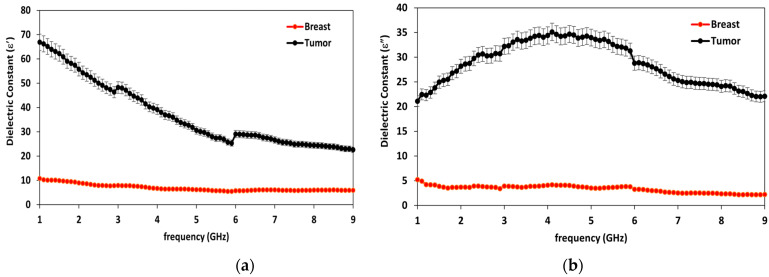
Measured electrical properties of breast phantom and tumor models versus frequency: (**a**) real part of dielectric constant (ε′) and (**b**) imaginary part of dielectric constant (ε″).

To verify the electrical properties of the breast and tumor models, SPEAG’s Dielectric assessment kit (DAK-3.5-TL2) at the microwaves measurements lab in the Electronics Research Institute (ERI) was used. Figure 4 presents the real and imaginary parts of the electric permittivity of the models. The real part (ε′) represents the dielectric constant (ε_r_), while the imaginary part (ε″) represents the losses.

### 2.4. Experimentals Setups

**Figure 5:**
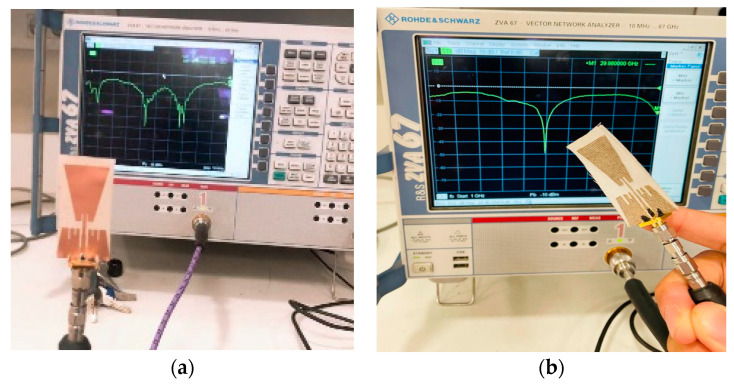
Full measurement setup of (**a**) flexible Roger substrate and (**b**) textile-based antenna connected to SMA cable at one side and other side to VNA.

This section presents all measurement setups used to evaluate the performance of the proposed antenna-based sensor in air and in proximity to models of the breast and tumors in frequency band up 10 GHz. For characterizing reflection and transmission properties, a two channel Rohde & Schwarz ZVA 67 vector network analyzer with frequency range up to 67 GHz is used as shown in Figure 5. Figure 5a shows the overall complete setup for flexible Roger substrate, while Figure 5b shows the textile antenna-based sensor connected to the cable during measurements.

### 2.5. Specific Absorption Rate (SAR) Measurements

**Figure 6:**
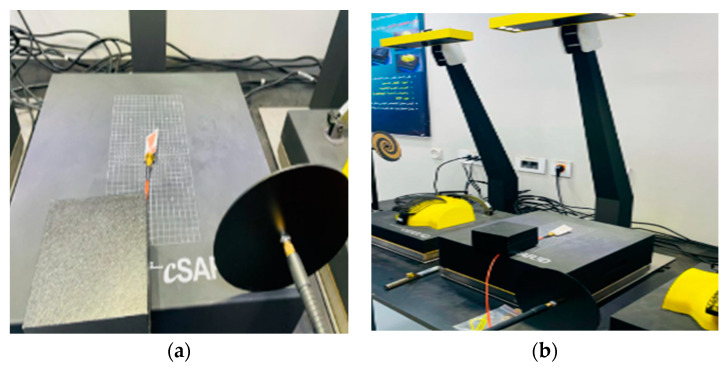
SAR measurements of the proposed antenna-based sensor: (**a**) flexible Roger substrate and (**b**) conductive textile.

To assess the safety of the wearable antenna-based sensors, specific absorption rate (SAR) is simulated and measured. A flat phantom for body-worn measurements (meets both IEC and FCC criteria for tissue-simulating media) as well as the left and right sides of the SAM head phantom are included in the equipment. Speag Switzerland is used for SAR measurements (cSAR3D) for conducting high-precision SAR measurements of devices operated on the body and both sides of the head as specified by IEC62209-1, IEEE 1528, FCC OET65, etc. cSAR3D performs fast (0.3 s) and repeatable (<0.1 dB) measurements in the band from 0.65 to 6 GHz [44]. Figure 6a,b show the SAR measurements complete setup for the flexible Roger substrate and textile-based antenna sensor, respectively.

### 2.6. Classification and Detection Algorithms

The proposed detection system is based on utilizing the deployment of different machine-learning algorithms to predict tumor in breast based on the effective relative permittivity of the breast tissues. This section provides an illustrative review on different machine-learning detection algorithms.

#### 2.6.1. Logistic Regression (LR)

In logistic regression (LR) technique, the prediction of a target variable is based on the fitting probability of the event on the logistic curve [45]. Logistic regression can be modeled as:

(1) $$\hat{Y}=\log\left(\frac{x}{1-x}\right)$$

where *x* is the probability of interested outcome and $\hat{Y}$ is the predicted output.

#### 2.6.2. Support Vector Machine (SVM)

**Figure 7:**
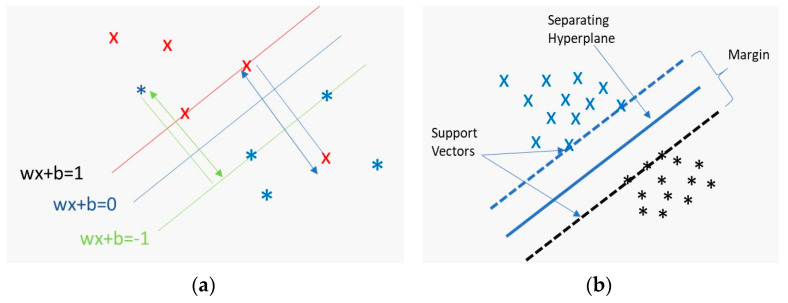
SVM classifier: (**a**) Projection of data points to compute the distance to a hyperplane [46]. (**b**) Illustration of the maximum margin between support vectors to separate between different classes [46].

Support vector machines (SVMs) are used for classification and regression tasks [45,46]. They are based on separating data of different classes using a separating hyper-plane the SVM can be calculated as follow:

(2) $$\begin{array}{c} \mathrm{If}\;Y_{i}=+1;wx_{i}+b\geq 1\\ \mathrm{If}\;Y_{i}=-1;wx_{i}+b\leq 1\\ \mathrm{For}\mathrm{all}i;\;y_{i}\left(wx_{i}+b\right)\leq 1 \end{array}$$

where *x* is a vector data point, *b* is a constant term that indicates the distance from the origin, and *w* is a weight vector as shown in Figure 7 [46]. The data [a] should always be greater than zero in order to separate it. The SVM selects the best hyper-plane where the distance between separated data is as large as possible. The optimum hyper-plane yields the maximum margin between closest points.

The SVM finds the hypothesis function, *f*(*x*) that separates different classes with maximum margins [46]. The SVM implements non-linear input vectors through mapping with a linear model. To tackle this problem, the SVM’s kernel function and hyper-parameters have to be properly defined. A proper setting of these parameters directly affects the model prediction performance [47].

Given an input training dataset, $\left(X,\;Y\right)=\left(x_{1},\;y_{1}\right),\;\left(x_{2},\;y_{2}\right),\cdots \;,\;\left(x_{N},\;y_{N}\right)$, the predicted output response ($\hat{y}$) of f(x) can be expressed as follows:

(3) $$\hat{y}=\langle \omega ,\phi \left(x\right)\rangle +b=\overset{N}{\underset{i=1}{\sum }}\left(\alpha_{i}k\left(x_{i},x\right)\right)$$

where the weight vector is denoted by *ω*, the feature vector is denoted by *ϕ*(*x*), *α_i_* are the support vectors coefficients, the non-linear feature kernel is *k*(*x_i_*,*x*), and *b* is a constant term. A polynomial kernel function is used to learn non-linear feature parameters as in [46]. The predicted output decision $\hat{y}$ is denoted as:

(4) $$\hat{y}=\langle \omega ,\phi \left(x\right)\rangle +b=\overset{N}{\underset{i=1}{\sum }}\left(\alpha_{i}{\left(\gamma x^{T}x^{\prime }+c\right)}^{d}\right)+b$$

#### 2.6.3. Decision Trees (DT)

**Figure 8:**
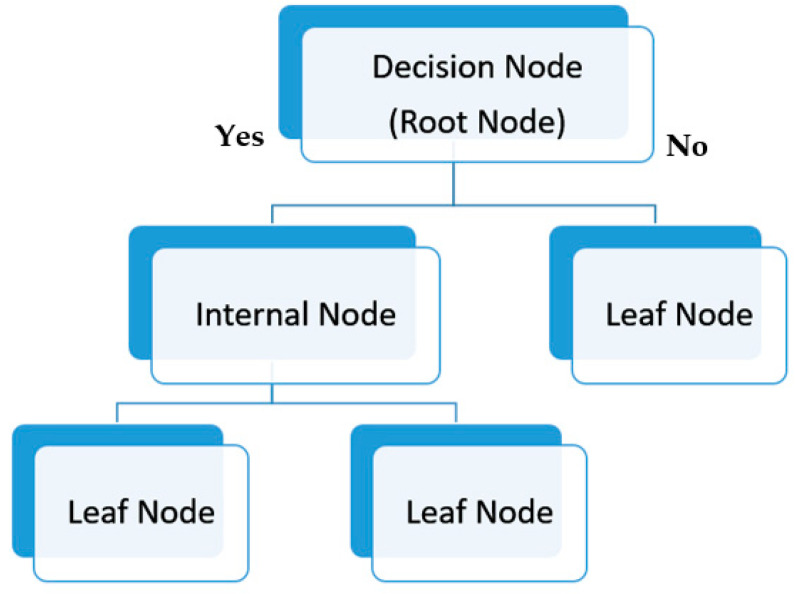
Illustrated example of binary decision tree.

Decision trees are used by many machine-learning applications due its ability for classification and prediction tasks [47,48,49]. A decision tree is a supervised learning algorithm based on a hierarchical structure for recursive splits of nodes with smaller steps. It consists of decision nodes and terminal leaves where the decision nodes are connected together by a predictive model and each leaf represents a class. The decision tree model is illustrated in Figure 8.

#### 2.6.4. Random Forest (RF)

Random forest is a supervised algorithm used by many researchers for classification tasks [50,51,52]. It uses ensemble learning where multiple classifiers are combined to solve complex problems. Random forest (RF) consists of different decision trees (DT) on various subsets of the given dataset. The decision of these trees is averaged to improve the predictive accuracy of that dataset. The RF algorithm utilizes ensemble voting of predictions to predict the final output instead of estimating prediction of a single decision tree.

#### 2.6.5. Gradient Boosting Methods (GBM)

Gradient boosting focuses on combining different decision trees as a weak learner to create a strong learner for accurate prediction [53,54]. It uses gradient descent as the optimization algorithm to minimize the loss function to obtain an improved learner. The gradient boosting for a specific loss function is illustrated as ψ(y, f) and for a base learner as h(x, θ) as mention in [53,54]. The algorithm yields $h\left(x,\;\theta_{t}\right)$ that is parallel to the negative gradient ${\left\{g_{t}\left(x_{i}\right)\right\}}_{i=1}^{N}$ of the data as mentioned in [53,54]:

(5) $$g_{t}\left(x\right)=E_{y}{\left[\frac{\partial \psi \left(y,f\left(x\right)\right)}{\partial f\left(x\right)}|x\right]}_{f\left(x\right)={\hat{f}}^{t-1}\left(x\right)}$$

It ends up with optimized least-squares solution as:

(6) $$\left(\rho_{t},\theta_{t}\right)=argmin_{\rho ,\theta }\overset{N}{\underset{i=1}{\sum }}{\left[-g_{t}\left(x_{i}\right)+\rho h\left(x_{i},\theta \right)\right]}^{2}$$

#### 2.6.6. Extreme Gradient Boosting (XGBoost)

Extreme gradient boosting (XGBoost) uses ensemble boosting for a decision tree algorithm [54,55]. The XGBoost combines multiple weak models to yield a better model. For different inputs and outputs, $\left(x_{1},y_{1}\right),\left(x_{2},y_{2}\right),\cdots ,\left(x_{n},y_{n}\right)$, the ensemble algorithm uses *K* additive functions to predict an output as mentioned in [54,55]:

(7) $${\hat{y}}_{i}=\overset{K}{\underset{k=0}{\sum }}f_{k}\left(x\right),\;f\in F$$

where f∈F is the space of CARTS. The function is approximated by minimizing regularized objective function for a given set of parameters θ as:

(8) $$obj\left(\theta \right)=\overset{n}{\underset{i=0}{\sum }}l\left({\hat{y}}_{i},y_{i}\right)+\overset{K}{\underset{k=0}{\sum }}\Omega \left(f_{k}\right)$$

where $l\left({\hat{y}}_{i},y_{i}\right)$ represents the training loss function of the predicted and real values, while $\Omega \left(f_{k}\right)$ denotes the regularization term that penalizes the model complexity as mentioned in [54,55].

#### 2.6.7. Light Gradient Boosting Machine (Light GBM)

Light gradient boosting machine (Light GBM) is a lightweight gradient boosting algorithm [56,57]. Light GBM is an accurate decision tree algorithm that produces complicated trees. It is based on the histogram algorithm where the data features eigenvalues that are converted into a histogram and the k bins intervals. Selecting appropriate parameters prevents overfitting of the model.

#### 2.6.8. Categorical Boost (“CatBoost”)

In this technique, both gradient boosting as well as categorical features are integrated together to yield the “CatBoost” algorithm [58,59]. It utilizes a random permutation and one-hot-max-size to emphasize categorical features to enhance algorithm robustness [59]. In “CatBoost”, a random permutation of the dataset is performed with an average label value assigned for each data sample [59]. In binary classification task, “CatBoost” boosts the classification accuracy with fast training speed.

## 3. Results

This section will be classified into three main subsections. The first section will represent both simulations and measurements for the textile-based antenna sensor in the free space. The second section will present both simulations and measurements of the proposed microwave antenna sensor while being placed on the breast phantom. Using breast phantoms in simulations and measurements will be performed in two scenarios: normal breast model and breast model with inclusion of tumor. The third section will represent the results of classification and detection techniques addressed in Section 2.5.

### 3.1. Characterization for Textile Antenna-Based Sensor

#### 3.1.1. Simulation Results

**Figure 9:**
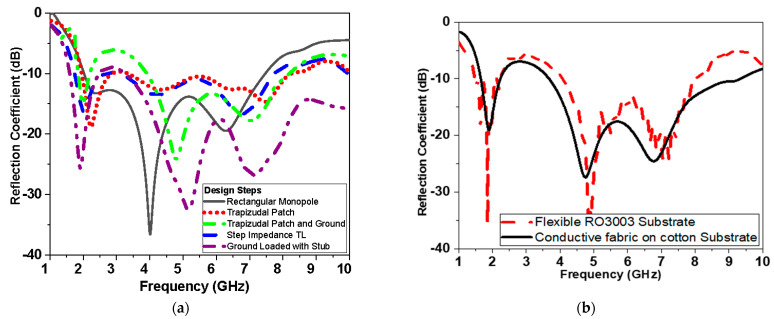
Simulated reflection coefficient in dB versus frequency: (**a**) different stages of monopole antenna design shown in Figure 1; (**b**) proposed monopole using flexible Roger 3003 and conductive textile fabric.

In this section, insights concerning the simulation and measurement results of the designed antenna sensor (CPW-based antenna) will be explored. The proposed monopole UWB antenna is simulated using HFSS v.15.0 based on finite element method (FEM). Moreover, a comparison of the performance of the antenna-based sensor using two types of conductors will be presented. The first conductor is flexible Roger as RT/Duroid 3003 substrate with height *h* = 0.13 mm, relative permittivity *ε_r_* = 3, with 0.0025 loss tangent, and the second is conductive fabric with sheet resistance of 0.5 Ω/□ as shown in Figure 9.

The effective dielectric constant ($\varepsilon_{reff\;}$) could be expressed by using Equation (19):

(9) $$\varepsilon_{reff}=\left(\frac{\varepsilon_{r}+1}{2}\right)+\left(\frac{\varepsilon_{r}-1}{2}\right){\left(1+\frac{12\times h}{w}\right)}^{-\frac{1}{2}}$$

The resonance frequency of the proposed rectangular monopole patch antenna could be calculated by using Equations (10) and (11), according to the standard formula [60].

The first resonant frequency ($f_{r}$) is:

(10) $$f_{r}=\frac{1.8411c}{2\pi D1{\left[\varepsilon_{reff}\left\{1+\frac{2h}{\pi L\varepsilon_{r}}\left(ln\left(\frac{L}{2h}\right)+\left(1.44\varepsilon_{r}+1.77\right)+\frac{h}{L}\left(0.258\varepsilon_{r}+1.65\right)\right)\right\}\right]}^{1/2}}≅\frac{c}{4*L*\sqrt{\varepsilon_{reff}}}$$

(11) $$L=L_{p1}+\left(L_{sub}-\left(L_{g}+L_{p1}+3.8\right)\right)\;\mathrm{mm}$$

where (c) is the velocity of light, ($f_{r}$) is the resonant of the frequency, *L_p_*_1_ is the length of the rectangular radiator patch, ($\varepsilon_{r}$) is the dielectric constant of the substrate, and h is the height of the substrate. So, by using the above equations $\varepsilon_{reff}=2.8$ and *L* = 25.5 mm, the first resonant frequency is about 2 GHz, and when the ground plane is modified and etched multiple slots the resonant frequency is reduced to 1.55 GHz.

**Figure 10:**
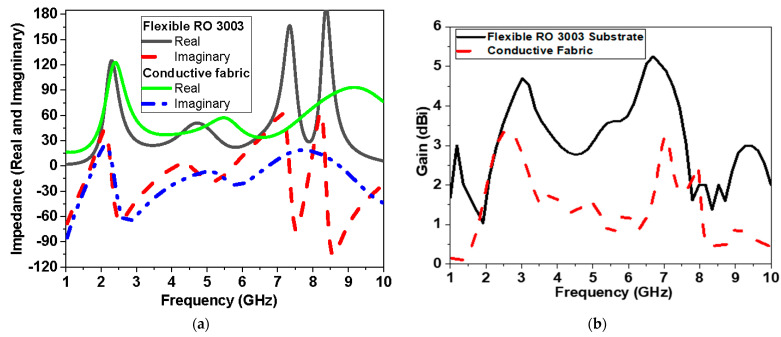
Simulation of proposed antenna-based sensor versus frequency using flexible Roger 3003 and conductive fabric: (**a**) real and imaginary part of impedance and (**b**) gain in dBi.

**Figure 11:**
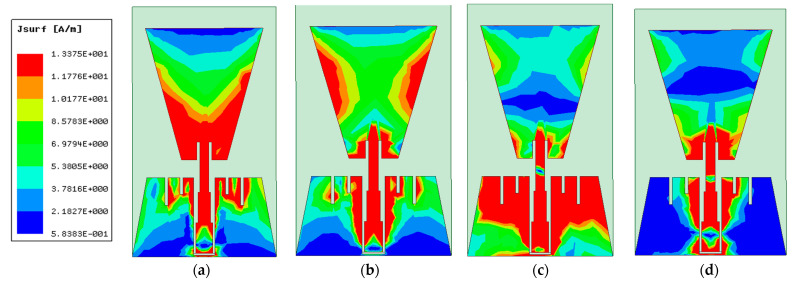
Current distribution at different frequencies: (**a**) 2.5 GHz, (**b**) 5 GHz, (**c**) 7.5 GHz, and (**d**) 10 GHz.

Both types of conductors reveal similar operation in terms of reflection coefficient, impedance matching (real and imaginary), and gain as shown in Figure 10a,b, respectively. The real and imaginary values of the impedance for both materials at frequency less than 6 GHz are almost the same as shown in Figure 10a. The gain shown in Figure 10b of flexible conductor substrate is higher than the textile antenna by 3 dBi on average over the operating proposed antenna band. Figure 11 shows the current distribution over the proposed antenna-based sensor at different frequencies over the operating band (2.5 GHz, 5 GHz, 7.5 GHz, and 10 GHz).

#### 3.1.2. Experimental Results

To validate the performance of the proposed antenna, the proposed antenna-based sensor is fabricated using two types of conductors tested in simulation models: flexible Roger substrate and conductive textile materials.

**Figure 12:**
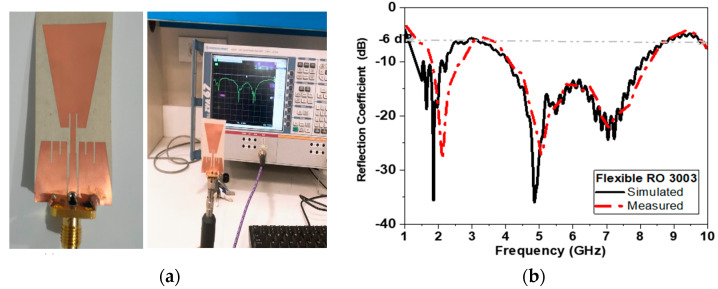
(**a**) Photo of fabricated antenna-based sensor using copper conductor tape and (**b**) measured and simulated reflection coefficient in dB versus frequency.

**Figure 13:**
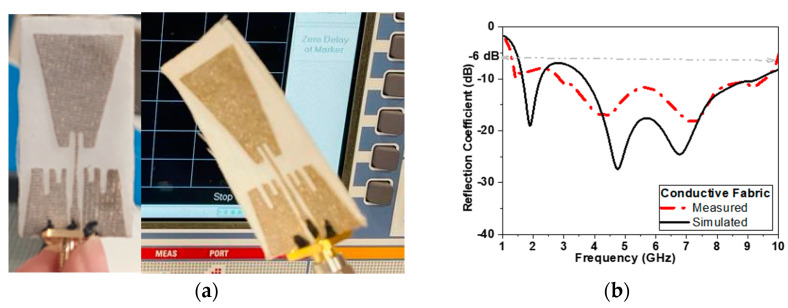
(**a**) Photo of fabricated antenna-based sensor using fabric conductor and (**b**) measured and simulated reflection coefficient in dB versus frequency.

Conductors are used for radiators and ground planes, while cotton is used for substrate as shown in Figure 12 and Figure 13. The antenna measurements are conducted using the VNR: 67 R&S^®^ ZVA VNA with frequency range up to 67 GHz (vector network analyzer) in the Electronics Research Institute (ERI) Labs. The SMA connector is connected by using carbon adhesion conductor epoxy with textile fabric conductor. Both figures show very good agreement between the measured and simulated results. This could be linked to the high quality of photolithographic shown in Figure 12, as well as high quality of textile fabrication by using laser cutting machine as shown in Figure 13.

### 3.2. Characterization for Textile-Based Antenna-Based Sensor with Breast Models

#### 3.2.1. Simulation Results: Reflection and Transmission Measurements

**Figure 14:**
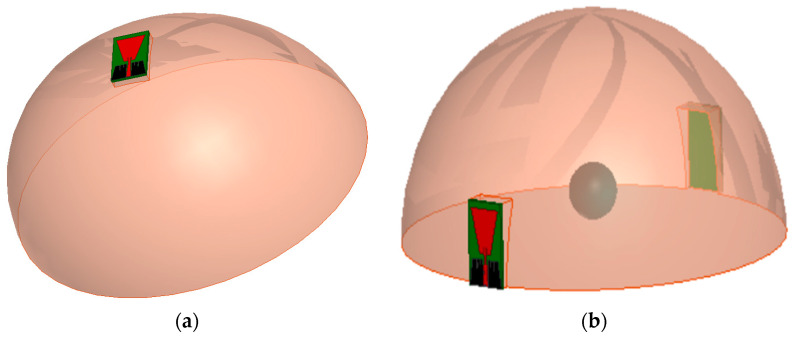
The layers model of breast with and without tumor tested using (**a**) one antenna and (**b**) two antennas.

To study the effect of the proposed antenna sensor on the body and its detection capability, a breast model is fabricated. The model is composed of one layer equivalent to the three main breast tissue layers of interest (skin, fat, and glandular tissue) [24,25]. It is slightly less accurate compared to three-layer models but significantly reduces the computation time. Two simulation scenarios were conducted: The first scenario use one antenna sensor in proximity to the breast phantom as shown in Figure 14a. The second scenario uses two antennas at both breast sides as shown in Figure 14b.

**Figure 15:**
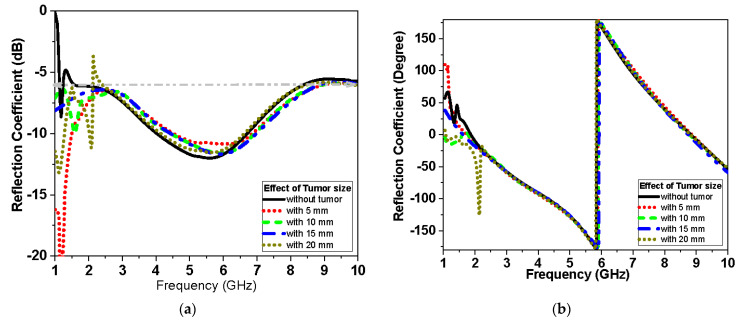
Simulated|S_11_|of proposed monopole flexible Roger antenna with tumor (first scenario as shown in Figure 14a) at different sizes of tumor: (**a**) magnitude in dB and (**b**) phase in degrees.

**Figure 16:**
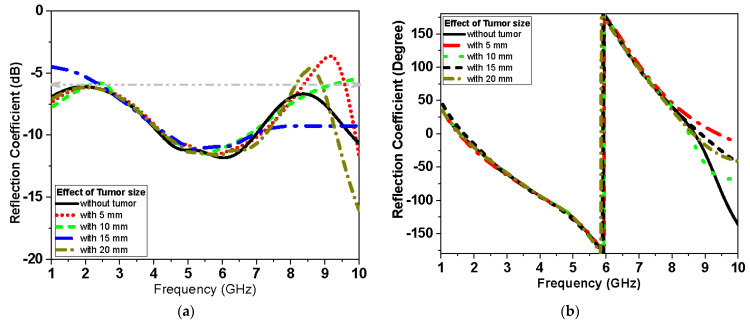
Simulated |S_11_| of proposed monopole textile antenna with and without tumor (second scenario shown in Figure 14b) at different sizes of tumor: (**a**) magnitude in dB and (**b**) phase in degrees.

**Figure 17:**
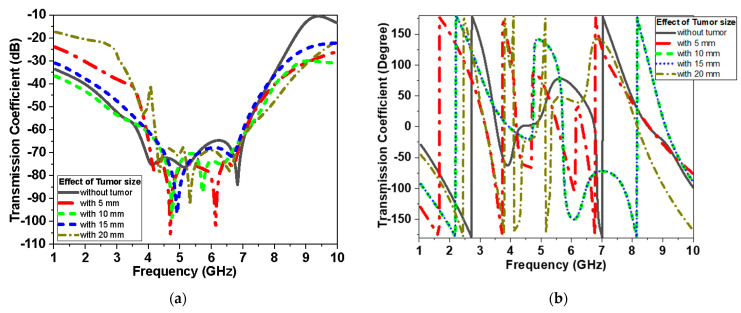
Simulated |S_21_| of proposed monopole antenna with and without tumor (second scenario shown in in Figure 14b) at different sizes of tumor: (**a**) |S_21_| magnitude in dB and (**b**) |S_21_|phase in degrees.

Two antennas configuration is used to further test transmission through the breast. The antenna is placed above the breast model with a buffer separation distance of 2 mm filled with cotton material. For the two scenarios, S-parameters with and without tumor are recorded as shown in Figure 15, Figure 16 and Figure 17. The parameters of the breast at 2.45 GHz are dielectric constant (ε_r_) = 11 F/m and conductivity (σ) = 2 S/m. The parameters of the tumor are dielectric constant (ε_r_) = 56 and conductivity (σ) = 5 S/m. Figure 14 presents the simulated |S_11_| magnitude and phase of the proposed monopole antenna without and with tumor at different sizes of tumor for the first scenario.

Figure 16 and Figure 17 present the magnitude and phase of the reflection coefficient and transmission, respectively, for the second scenario. The reflection coefficient of Figure 15, Figure 16 and Figure 17 is less sensitive at small tumor size less than 10 mm. While the tumor size increases, the S_11_ becomes more sensitive and the variation of S_11_ values becomes noticeable, especially at lower frequency less than 2.5 GHz band and higher frequency larger than 7 GHz band for the first and second scenario, respectively. Figure 17 shows the effect of tumor size on the transmission coefficient coupled between the two antenna elements over the operating band at different tumor sizes.

#### 3.2.2. Experimental Results: Reflection and Transmission Measurements

**Figure 18:**
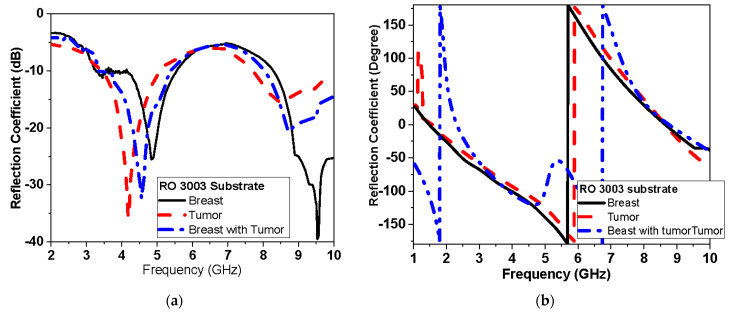
Measured |S_11_| of flexible Roger substrate antenna performance with breast phantom and tumor: (**a**) magnitude and (**b**) phase.

**Figure 19:**
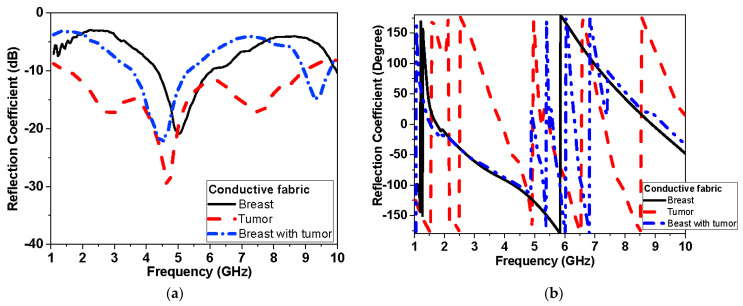
Measured |S_11_| of conductive fabric antenna performance with breast phantom and tumor: (**a**) magnitude and (**b**) phase.

**Figure 20:**
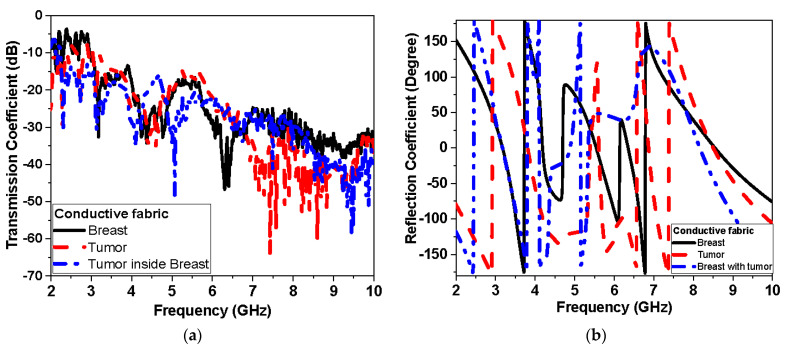
Measured S21 of conductive fabric antenna performance with breast phantom and tumor: (**a**) magnitude and (**b**) phase.

The response of the reflection coefficient of the proposed antenna sensor using different conductors used flexible Roger and textile fabric conductor as shown in Figure 18 and Figure 19, respectively. Figure 18 shows the measured proposed monopole|S_11_| magnitude and phase of flexible Roger substrate antenna performance in free space and applied to the tumor and breast with tumor. Figure 19 and Figure 20 show the measured proposed monopole|S_11_| and |S_21_| magnitude and phase of the textile antenna performance, respectively, in free space and when applied to the tumor and breast with tumor.

#### 3.2.3. SAR Measurements

The specific absorption rate (SAR) can determine how much power of these radiations the human tissues absorb. The antenna can be called safe if its max SAR value does not exceed 1.6 W/kg [39,44]. As a guide, the patient should not be exposed to any health hazards because a mobile phone uses the same frequency band and microwave breast cancer imaging uses less radiation than a mobile phone. The measurements are also made at the central laboratories in the Electronics Research Institute using a special device for measuring SAR at different power levels for both antennas. Table 2 and Table 3 show the measured SAR levels for the two types of proposed monopole antenna (Roger substrate and textile) at 2.45 GHz and 5.2 GHz, respectively.

### 3.3. Detection Results

In this section, two experimental examples have been carried out to compare between different machine-learning classification algorithms for breast tumor detection. These experiments utilized the |S_11_| and |S_21_| parameters, and radial antenna in detection and classification of the tumor.

These examples verify the importance of the $S_{21}$ parameter as an additional degree of freedom in increasing the classification accuracy. Moreover, the classification accuracy is influenced by the tumor size as well as number of features. The results demonstrate the sensitivity of the machine-learning algorithms for the detection tumors in the form of class testing accuracy.

#### 3.3.1. Dataset

The dataset samples are created using simulated S-parameters in two scenarios. The first scenario utilizes one antenna-based sensor placed above the breast nipple model and the tumor is placed in the middle of the breast at a distance of 60 mm as shown in Figure 14a. The tumor size is increased in the study from 5 mm to 10 mm and 20 mm, respectively, as shown in Figure 15. It is worth mentioning that the tumor size of 5mm has no noticeable effect. Therefore, the developed dataset is chosen to have 10 mm as well as 20 mm tumor size. In the first scenario dataset, the tumor is detected using single microwave antenna sensor with reflection coefficient parameter features (|S_11_| magnitude and phase) as well as axial antenna for each 10 mm and 20 mm tumor size. The data samples are 366 samples associated with their labels for each tumor size detected over frequencies 2 GHz–8 GHz. The data is balanced for benign and malignant tumor cases.

In the second scenario dataset, two antenna-based sensors are utilized at different sides of the breast model as shown in Figure 14b, with different sizes of tumor as shown in Figure 16 and Figure 17. The size of the datasets are equal to the number of frequency points selected. The second dataset features parameters of both (|S_11_| and |S_21_| magnitude and phase) as well as axial antenna for 10 mm and 20 mm tumor size. The data samples are 183 samples associated with their labels for each tumor size detected over frequencies 2 GHz–8 GHz. The labels were encoded into 0, 1, and 2, representing ‘No Tumor Found’, ’10 mm Tumor found’, and ‘20mm Tumor found’, respectively. The dataset samples are divided into two parts; training data and testing data. The training data are fed to multiple machine-learning models to train, and then tested on the testing dataset.

#### 3.3.2. Preprocessing

Feature scaling and stratified sampling were used to scale the data samples’ features and split the data into training and testing datasets. Mean normalization was used as a data normalization technique to normalize the data features. Stratified sampling was used as a data separator in splitting the data into training and testing data along with shuffling the samples to ensure that all the data categories are represented equally in the testing phase.

(12) $$X_{\mathrm{Stand}}=\frac{x-\mathrm{mean}\left(x\right)}{\mathrm{standard}\;\mathrm{deviation}\;\left(x\right)}$$

#### 3.3.3. Evaluation

The evaluation metric used for verifying the performance of the classification algorithms is the accuracy which is defined as the total number of correctly classified patterns divided by the total numbers of patterns:

(13) $$\mathrm{Accuracy}=\frac{\mathrm{TP}+\mathrm{TN}}{\mathrm{TP}+\mathrm{FP}+\mathrm{FN}+\mathrm{TN}}$$

where TP, TN, FP, and FN represent true positive, true negative, false positive, and false negative, respectively:

1. True negative: the observation is correctly classified as negative.
2. False negative: the observation is incorrectly classified as negative.
3. True positive: a positive class is correctly classified by the model.
4. False positive: a negative observation is incorrectly classified.

**Figure 21:**
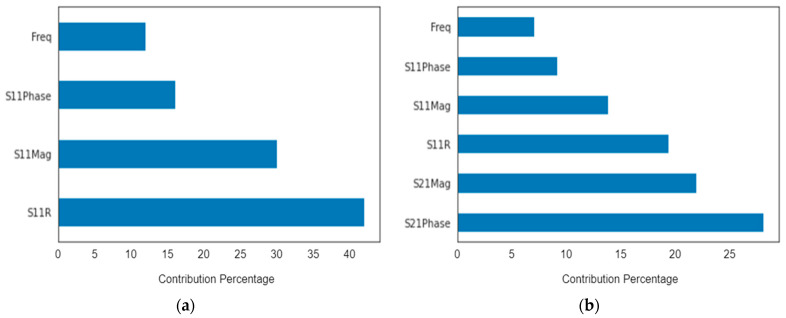
Contribution of each feature parameter for the classification accuracy of the “CatBoost” algorithm (**a**) for first dataset of first scenario and (**b**) for second dataset of second scenario.

To verify the model evaluation performance, a confusion matrix is used for representing the ability of the model to classify labels correctly. The confusion matrix describes the performance parameters for the classifier. The training data is fed to multiple machine- learning models to train, and then tested on the testing dataset as shown in Table 4. This table shows the sensitivity of each algorithm towards each class. In the first dataset, where $S_{11}$ magnitude and phase parameters are considered, the “CatBoost” algorithm has the highest accuracy with 67% and 69% for 10 mm and 20 mm tumor sizes, respectively. In the second dataset, where the number of data featured is increased by adding $S_{21}$ magnitude and phase, it is clearly shown that the classification accuracy has greatly improved to 83% and 100% for 10 mm and 20 mm tumor sizes, respectively, as shown in Table 5 and Figure 21.

#### 3.3.4. Feature Importance

Feature importance illustrates the impact of training features regarding the prediction target, quantifying the effectiveness of the relevant features against the predicted decision. The table below shows each feature among the two conducted experiments and its exact percentage of contribution, introducing S_11_ parameters with magnitude and phase antenna along with the effect of the addition of S_21_ parameters. The figure below represents the importance of comparison of the data samples’ features. It verifies the strength of each data samples’ features.

## 4. Discussion

This paper presents the development of a complete wearable breast cancer detection and monitoring system based on flexible antennas acting as sensors in the microwave band. The study in this paper develops compact and ultra-wideband flexible sensors using two technologies. The first technology used is flexible Roger substrate while the second is conductive fabric implemented on cotton substrate. The performance of the proposed antenna-based sensor using the two technologies is recorded and compared. The simulations validate similar performance of the proposed antenna-based sensor antenna using conductor fabric compared to flexible Roger substrate, especially at frequency less than 7 GHz. Both types of conductors reveal similar operation in terms of reflection coefficient and impedance matching. The wearable textile antenna sensors acquire UWB performance with extended bandwidth from 1.6 GHz up to 10 GHz at |S_11_| ≤ −6 dB and acceptable antenna average gain of about 3 dBi. The gain using flexible conductor substrate is higher than the textile antenna by 3 dBi on average over the operating antenna band. The sensors fabricated with conductive fabric show better performance compared to those with flexible Roger substrate sheet as it provides lower substrate loss and better fitting with women’s breasts where there is no air in between. On the other hand, the sensor fabricated using flexible copper surface interacts with human sweat and could be subjected to rusting over time. Thus, conductive fabrics are more favored for fabricating wearable sensors in this study.

This system relies on electromagnetic microwave technology to detect differences in electrical properties by measuring magnitude and phase of reflection and transmission coefficient between normal breast tissue and tumor-affected tissue. This is highly shown in terms of detection tumors in measurements and simulations results. Two testing scenarios have been proposed in this paper. The first scenario uses one sensor over breast phantoms, while the second scenario uses two sensors. The second scenario allows collecting transmission data (S_21_) in addition to reflection (S_11_) as the first scenario. Each S-parameter adds two parameters, the magnitude and the phase, for detection dataset data. Simulations show that the reflection coefficient magnitude and phase difference increased by increasing the tumor size, especially within the band from 1 GHz to 3 GHz for the first scenario. In addition, for the second scenario, the reflection coefficient magnitude and phase difference increased as the tumor size increased, especially within higher frequency of operation larger than 7 GHz. Transmission coefficient (S_21_) shows high sensitivity in detection over the operating band. Simulation studies have been conducted using the effective model of the breast (one layer with effective dielectric constant (ε_r_ = 11) representing skin, fat, and glandular tissues of the breast. The given model succeeds in showing the change in response by changing the size of the tumor even with matching level −6 dB for reflection coefficient while being on the breast. Figure 15a and Figure 16a record the changes in reflection coefficient at matching levels of −6 dB. The proposed system is very good in detecting the malignant tumor in the second and third stage.

Furthermore, breast and tumor phantoms have been fabricated and characterized using DAK-3.5-TL2 (dielectric probe station) in the range of 1–9 GHz. Figure 4 shows that there are distinguished differences in electrical properties between the breast and the tumor models over the operating band extended from 1 GHz to 9 GHz. The average real part of the breast phantom ε′_r_ is about 7, while the tumor ε′_r_ is about 55. Moreover, the imaginary part of the breast phantom and tumor ε″_r_ is about 4 and 27, respectively. This validates sensing tumor tissue inside the breast with measured contrast ratio 1:8. The proposed sensors have been placed on fabricated phantoms and measured using a vector network analyzer (VNA). Variations have been recorded in both reflection (S_11_) and transmission (S_21_) as shown in Figure 18, Figure 19 and Figure 20 when tumor phantom is inserted inside the breast model.

The proposed antenna-based sensors measured low SAR values below 1.6 W/kg at different operating frequencies (2.45 GHz and 5.2 GHz). The antenna-based sensor using conductive fabric realized lower SAR compared to flexible Roger substrate at different power levels as shown in Table 2 and Table 3. The antenna-based sensor using conductive fabric realized lower SAR of 0.55 W/kg at 1g compared to flexible Roger substrate with SAR level of 1.24 W/kg at 25 dBm power level. The given low SAR level validates the safety of the proposed antenna sensor as a wearable device. It also allows future implementation of multiple antenna sensors for the required detection system.

Moreover, machine-learning algorithms have been developed and used to classify different scanning states using recorded S-parameters. The simulation examples also verify the capabilities of machine-learning algorithms in breast cancer tumor classification. A comparison between different classification techniques has been carried out for different datasets of different tumor sizes. These examples verify the effectiveness of the $S_{21}$ parameter in improving the classification accuracy of “CatBoost” from 69% to 100% for 20mm tumor size. It has also shown that the classification accuracy is influenced by the tumor size, in which the accuracy improved from 83% to 100% between 10 mm and 20 mm tumor sizes.

Table 6 compares the performance of the proposed flexible antenna-based sensor using conductive fabric with other designs presented in the literature for breast cancer. Table 6 shows a comparison for only systems that use microwave signal for breast cancer detection and imaging. It could be noted that most of the published work used the rigid FR4 substrate compared to the proposed flexible substrate in this study for sensor fabrication. Flexible substrates allow conformal and comfortable placement on the human body and are more appealing in terms of developing wearable systems. Most of the antenna-based sensors are of type Vivaldi with end-fire operation. Systems based on Vivaldi antennas will limit the detection system fixed in the CT room and are very hard to develop as self-screening systems. Moreover, most of the published work acquired limited bandwidth and some did not study or record SAR values as shown in Table 6. SAR value is highly important for sensors intended to be in proximity with the human body.

To compare the performance of different sensors in the literature with the proposed sensor in this paper, the figure of merit of antenna-based sensor (*FOM_A_*) equation developed in [60] will be used. The given equation is used to compare the performance of planar sensors in biomedical applications. From [61], the overall performance of a planar sensor is improved when the *FOM_A_* is increased. Using the given equation to compare the monopole antennas in Table 6 acting as sensors, our proposed sensor realizes −15 dB compared to −51.67 dB in [64] and −37.38 dB in [61].

The proposed system is implemented using fully conductive fabric with low SAR value, which makes the system safe, portable, and easily wearable as a “Smart Bra”. This proposed system has the potential to be a great addition to women’s health care. The proposed system will provide a user-friendly, in-house, pain-free, low-cost scanning system for women who require continuous examination of their breasts.

## Figures and Tables

**Table 1:** The optimized dimensions of the proposed antenna sensor (all dimensions in mm).

Antenna Sensor and Substrate	Feed Line	Ground
W_sub_ = 24	W_p2_ = 7.5	L_s1_ = 5
L_sub_ = 45	L_p2_ = 20	L_s2_ = 3
W_p1_ = 20	W_f_ = 2.8	L_g_ = 14.25
L_p1_ = 23.75		

**Table 2:** Comparison of measured SAR levels for the proposed monopole antenna at 2.45 GHz.

Flexible Monopole Antenna		Textile Monopole Antenna		Power Level
10 g	1 g	10 g	1 g	(dBm)
0.035 W/kg	0.172 W/kg	0.034 W/kg	0.073 W/kg	5
0.110 W/kg	0.332 W/kg	0.110 W/kg	0.232 W/kg	10
0.223 W/kg	0.463 W/kg	0.123 W/kg	0.263 W/kg	15
0.32 W/kg	0.657 W/kg	0.125 W/kg	0.267 W/kg	20
0.75 W/kg	1.24 W/kg	0.25 W/kg	0.55 W/kg	25

**Table 3:** Comparison of measured SAR levels for the proposed monopole antenna at 5.2 GHz.

Proposed Monopole Antenna Copper		Proposed Monopole Antenna Textile		Power Level
10 g	1 g	10 g	1 g	(dBm)
0.010 W/kg	0.039 W/kg	0.010 W/kg	0.039 W/kg	5
0.018 W/kg	0.054 W/kg	0.016 W/kg	0.065 W/kg	10
0.115 W/kg	0.280 W/kg	0.036 W/kg	0.088 W/kg	15
0.174 W/kg	0.542 W/kg	0.115 W/kg	0.280 W/kg	20
0.547 W/kg	1.70 W/kg	0.3 W/kg	0.624 W/kg	25

**Table 4:** Classification accuracy of different algorithms for both datasets.

	|S_11_| + Phase				|S_11_| + |S_21_| + Phase			
Classes	No Tumor	10 mm	20 mm	Total	No Tumor	10 mm	20 mm	Total
Logistic Regression	33%	50%	38%	40%	67%	17%	43%	31%
Support Vector Machine	33%	50%	46%	43%	33%	17%	29%	26%
Decision Tree	42%	58%	54%	51%	67%	50%	100%	73%
Random Forest	58%	58%	31%	48%	67%	17%	43%	42%
LightGBM	50%	58%	46%	51%	50%	100%	57%	68%
Kneighbors	58%	42%	54%	51%	67%	33%	57%	52%
XGBboost	42%	67%	54%	54%	67%	83%	43%	63%
AdaBoost	33%	50%	38%	40%	67%	33%	57%	52%
CatBoost	42%	67%	69%	59%	83%	83%	100%	89%

**Table 5:** Feature importance of each parameter in the two datasets for the “CatBoost” algorithm.

	|S_11_| + Phase	|S_11_|+ |S_21_| + Phase
Frequency	14.0%	7.0%
S_11_ Phase	14.8%	9.1%
S_11_ Magnitude	28.2%	13.9%
S_11_ Axial	42.9%	19.5%
S_21_ Phase	-	28.3%
S_21_ Magnitude	-	22.2%

**Table 6:** Comparison of proposed antenna and others reported in the literature (* work presented in the manuscript).

Ref.	Antenna Type	Size mm^3^	$\mathrm{Ag}\;(\lambda_{g}^{2})$	Flexible	Operating Bandwidth GHz	Efficiency η %	Imaging Method	Gain (dBi)	SAR (W/kg)	Wearable
[21]	Monopole	30 × 30 × 0.05	0.22 × 0.22	Yes	2–4	NM	NM	NM	1.6	Yes
[61]	Monopole	13 × 13 × 0.0125	0.35 × 0.35	Yes	7 to 14	65	NM	4.4	NM	Yes
[62]	Vivaldi	40 × 40 × 1.6	0.4 × 0.4	No (FR4)	2.5–11	77	MERIT	7.2	NM	No
[63]	Monopole	30 × 30 × 0.1	1.09 × 1.09	Yes	5.71–5.99	80.5	NM	3.08	0.174	Yes
[64]	Vivaldi	49 × 46 × 0.8	1.1 × 1	No (FR4)	3.1–10.6	NM	DMAS	7.5	NM	No
[65]	Vivaldi	51 × 42 × 0.05	0.8 × 0.65	No (Roger 5870)	2.8–7	70	IC-DAS	7.5	NM	No
[66]	Vivaldi	25 × 20 × 0.1	0.58 × 0.47	No (Polyamide substrate)	3.8–4 & 8–10	NM	MERIT	2.33	NM	Not
Our	Monopole	24 × 45 × 0.17	0.38 × 0.2	Yes	1.8–10	70	CatBoost	3.5	0.58	Yes

NM: not mentioned; IC-DAS: iteratively corrected delay and sum; MERIT: microwave radar-based imaging toolbox; DMAS: delay multiply and sum; MLA: machine-learning algorithms.

## Data Availability

Not applicable.
